# Lexical Variation and Change in British Sign Language

**DOI:** 10.1371/journal.pone.0094053

**Published:** 2014-04-23

**Authors:** Rose Stamp, Adam Schembri, Jordan Fenlon, Ramas Rentelis, Bencie Woll, Kearsy Cormier

**Affiliations:** 1 Deafness, Cognition and Language Research Centre (DCAL), University College London, London, United Kingdom; 2 Linguistics program and The Centre for Research on Language Diversity, La Trobe University, Melbourne, Australia; The University of Chicago, United States of America

## Abstract

This paper presents results from a corpus-based study investigating lexical variation in BSL. An earlier study investigating variation in BSL numeral signs found that younger signers were using a decreasing variety of regionally distinct variants, suggesting that levelling may be taking place. Here, we report findings from a larger investigation looking at regional lexical variants for colours, countries, numbers and UK placenames elicited as part of the BSL Corpus Project. Age, school location and language background were significant predictors of lexical variation, with younger signers using a more levelled variety. This change appears to be happening faster in particular sub-groups of the deaf community (e.g., signers from hearing families). Also, we find that for the names of some UK cities, signers from outside the region use a different sign than those who live in the region.

## Introduction

**Figure 1:**
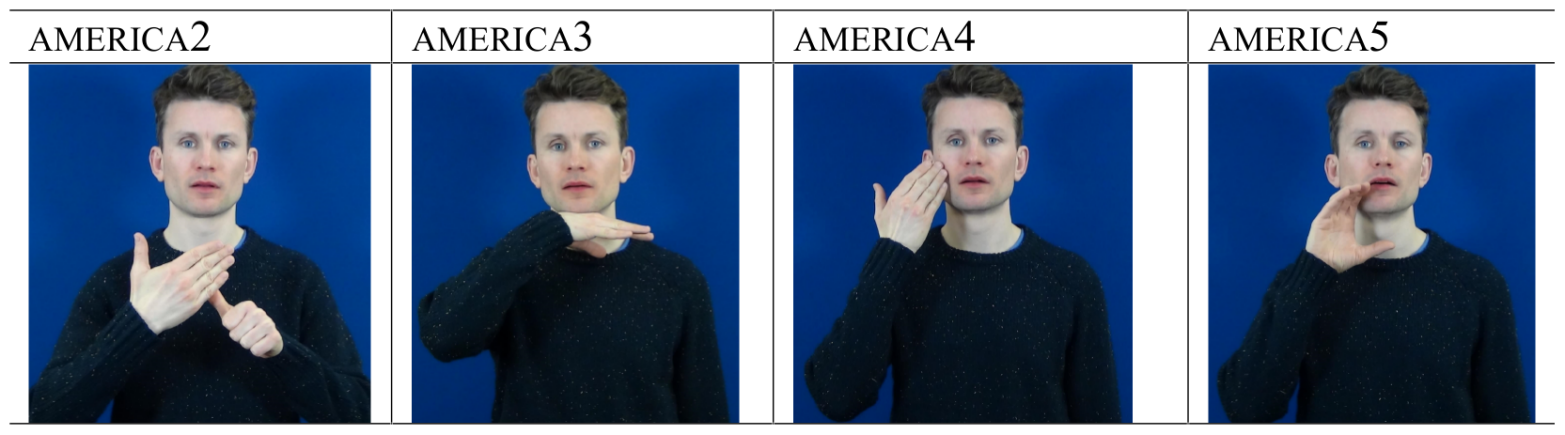
Four regional lexical variants for the concept ‘America’ in BSL.

Variation is an intrinsic part of all languages whether spoken or signed. It is apparent at all levels of language organisation: for example, there are several lexical variants in British Sign Language (BSL) which all mean ‘America’ (see Figure 1) (following the glossing convention used in sign language literature, examples of sign variants are represented by a corresponding English word written in small capitals, e.g., monday; lexical variants, which have the same meaning, are represented with numbers following the gloss, e.g., monday, monday2 - as outlined by Cormier, Fenlon, Johnston, Rentelis, Schembri, et al. in 2012, [1], the gloss used in this paper reflects the glossing system used in the BSL Corpus Project and the BSL lexical database arising from it). Similar lexical variation has been observed in various sign languages studied to date, including American Sign Language (ASL) [2] and newly emerged sign languages, such as Nicaraguan Sign Language [3]. Variation in lexis may be systematically used by speakers to index their affiliation with particular social groups [4]. Recently, work has shown that this is also true for sign languages [2]. This variation may function as an index of social variables such as region, gender, ethnicity, and social class, or social factors that are distinctive to sign language communities [2], such as the language policy of the school attended during childhood or the language background of the signer's family. It may also be indicative of a language change in progress [5].

In an earlier study, sociolinguistic variation and change in BSL numeral signs was investigated. Stamp, Schembri, Fenlon and Rentelis [6] found that lexical variation in signs for the numerals 1 to 20 was systematically constrained by several social factors. Age, school location and language background (whether a signer is from a deaf or hearing family) were found to be significant predictors of a signer's lexical choice. Younger signers used a decreasing proportion of regionally distinct number signs, suggesting that levelling may be taking place. It had previously been suggested that this change may be a result of increased dialect contact [7] and increased exposure to regional variants through the media [8]. Stamp et al.'s study [6] formed part of the larger investigation of lexical variation undertaken as part of the BSL Corpus Project [9]. In this paper, we look at sign variants for colour terms, foreign and UK place names as well as numerals in data from a lexical elicitation task. Variants for these four semantic categories were elicited from 249 deaf native, near-native and early learner BSL users recruited from eight UK cities (we use the term ‘native signers’ here to refer to those individuals who acquired BSL from birth, ‘near-native signers’ as those who acquired the language before beginning school, and ‘early learners’ to refer to deaf adults who report acquiring BSL during primary school). Forty-one lexical items are analysed and correlated with the semantic category of the sign and the following social factors: region, age, gender, social class, language background and school location. The sign variants produced for colours, countries and numbers were coded as either ‘traditional’ or ‘non-traditional’ for the signer's region of residence in order to determine whether there is empirical evidence for *levelling* – i.e. the reduction in use of regionally marked variants which is thought to be the outcome of regular face-to-face interactions between speakers of differing linguistic repertoires [10]–[11]. Anecdotal reports from the deaf community indicate that BSL may be undergoing levelling, given previous suggestions of change in the past thirty years because of increased exposure to lexical variants through the media [8],[12]–[13]. For UK place names, we were interested in finding evidence related to anecdotal reports that signers use different variants depending on their in-group and out-group status (i.e., residents of a specific urban centre use a different sign to refer to their city from the one used by non-residents). The most comparable research on lexical variation to date was conducted on New Zealand Sign Language (NZSL), a sign language closely related to BSL [14]. McKee and McKee [14] found that variation in the NZSL lexicon had become increasingly levelled following the introduction of the Australasian Signed English system (from Australia) into New Zealand deaf education, with younger signers making greater use of the lexical variants associated with this system. The research aims of the current study are to investigate: (1) the extent of lexical variation in the target lexical items in BSL; (2) the degree to which this variation reflects social factors such as age, region, gender and language background; and (3) whether there is evidence for a language change in progress, including possible levelling in BSL.

This paper is organised into five parts: first, we discuss the history of BSL regional variation. Next, we present a brief review of the literature on spoken and signed language lexical variation. We then explain the methodology for the BSL Corpus Project and present the results of this study. Finally, we discuss the findings in relation to other relevant sign language studies and consider their implications for an understanding of variation in BSL.

### The British deaf community

The 2011 Census for England and Wales reports that between 15,000–20,000 people in the UK use BSL as their main language [15]. BSL, the sign language of the British deaf community, is also closely related to the sign languages used in Australia (Auslan) and New Zealand (NZSL), with some researchers even suggesting that they are dialects of the same sign language: BANZSL (British-Australian-New Zealand Sign Language) [16].

#### Schools and the development of BSL regional variation

Typically language is transmitted from caregiver to child. However, the vast majority of deaf children are born to hearing parents (Mitchell & Karchmer [17] found 92% of deaf children in the USA have two hearing parents; Uus & Bamford [18] found 10.6% of congenitally deaf children in the UK have a family history of deafness). Hearing parents are unlikely to know a sign language, and a deaf child may have limited access to the spoken language. Acquisition of a first language may thus be delayed for many deaf children [19]–[20]. Schools for deaf children, especially residential schools, have therefore long been associated with sign language transmission [21]. Since the first deaf schools were opened in 1760, sign language has been used as a form of communication among deaf children and passed on from older to younger peers [22]. Because there was minimal interaction between schools and no standard or written form of BSL, these ‘school-lects’ continued to develop separately in each community [13]. It is believed that deaf school-leavers maintained the use of these school variants in the local community and they became the basis for current regional varieties of BSL [13],[23]. These regional varieties have been found to vary most obviously at a lexical level (although see Fenlon, Schembri, Rentelis, & Cormier [24] for a study on phonological variation from the same regions reported here).

#### Changes in the British deaf community

In recent years, anecdotal claims within the British deaf community suggest that traditional regional variation is in decline, and there is some evidence for these claims, e.g., in numeral signs [6]. Several recent changes in the British deaf community have been offered as possible causes. Perhaps the most important of these changes has been the closure of centralised schools for deaf children. In the late 1970s, the Warnock Report recommended that deaf children be sent to integrated schools alongside their hearing counterparts [25]. As a result, many residential schools for deaf children closed [26], and deaf children have been increasingly sent to mainstream schools [21]. Centralised schools for deaf children, which would have once served as an opportunity for deaf children from hearing families to acquire signing from their native signing peers [23], are being replaced by the mainstreaming of deaf children where hearing educational and communication support workers enable deaf children to participate in classroom activities alongside hearing children. In the absence of deaf peers, these communication support workers sometimes serve as language models for the children, despite the fact that they may have limited sign language skills themselves [27]–[30].

In addition, increased mobility and transnational contact have exposed deaf and hearing British people alike to a multitude of languages, dialects and social practices. Research on British English accents has shown how increased mobility within the UK has resulted in dialect levelling [31]–[32]. Whilst levelling has not been widely researched in sign languages, there is evidence that increased international interaction has influenced the lexicon of a number of sign languages [33]–[34]. Advances in technology such as the use of webcams and online video have substantially increased exposure to BSL signers outside an individual's local community. Broadcast media have had an impact on the lexicon, with younger signers reported to incorporate new signs seen on television into their BSL [8], and the suggestion that some Scottish regional signs have gained more widespread currency through the influence of Scottish presenters on the BBC deaf community programme *See Hear* [12].

Changes in BSL resulting from the emergence of TV programmes for the deaf community and sign language interpreting on television may have led to an increased preoccupation with political correctness since the 1990s. Signs for foreign countries which portray physical features have sometimes been perceived as ‘racist’ by the hearing non-signing community [35]–[36]. As a result, it has been claimed that traditional BSL signs meaning ‘China’, ‘Africa’, ‘gay’ and ‘India’, for example, may have become less commonly used by younger signers because of concern that their form was strongly associated with stereotypical images or actions associated with these groups [12]–[13]. However, some of the country name signs in the lexicon appear to be changing for reasons that cannot be attributed to political correctness (e.g., the traditional variants meaning ‘America’ cannot be considered offensive). Thus, political correctness alone cannot explain all the patterns of lexical change, even within this specific semantic domain.

#### Lexical variation in signed and spoken languages

Sociolinguistic research has identified the following social factors as providing important insights into the nature of language variation and change in spoken and signed languages: age [14],[37], gender [38]–[40] and social class [2],[41]. Age-related variation in lexis has been reported for BSL [42]. As a broad generalisation, for example, older deaf people of the late 1980s used more fingerspelling (the use of a manual alphabet to spell out English words) than younger deaf people of the late 1980s, reflecting previous educational practices (a more recent study indicated this is also true of Auslan, see [43]). Iconicity as a factor in sign creation may also result in age differences. As new technology has replaced old, lexical items used by younger signers may reflect the changed appearance or means of operating new appliances, while older signers may maintain the sign in its earlier form [44]. For example, an earlier sign meaning ‘telephone’ represented how a person would hold a candlestick phone whilst a newer variant resembles how a person holds a mobile phone.

Although sociolinguistic investigations have tended to concentrate on variation and change in the phonology and grammar, lexical variables are also an important point of sociolinguistic investigation [45]–[50]. Nevertheless, it has been argued that the lexicon cannot tell us anything about language change, since speakers continually adopt new concepts into their vocabularies [51]–[53].

In contrast to the focus in contemporary studies in the sociolinguistics of spoken languages on sociophonetic variation, the obvious presence of considerable variation in sign language lexicons has meant that studies of variation in sign language have emphasised lexical variation and change. In contrast to spoken languages, regional and social sign language ‘accents’ have not been described, although some subtle variation in the application of phonological processes to specific sublexical elements does appear to be correlated with region [2],[24],[54].

Theories of regional dialects often implicitly presuppose that there was once a single, uniform language, which diverged until identifiable regional varieties arose, either through spontaneous evolution or language mixing, or both processes [55]. There is no evidence, however, that there was once a single variety of BSL, which split up as deaf people spread throughout the country. We can also probably dismiss the idea that regional variants in BSL differ primarily because of mixing with other languages (although there is evidence that some Scottish and Northern Irish varieties have been influenced by Irish Sign Language and American Sign Language, see [56]–[57].

Woll et al. [58] identified considerable lexical variation between the varieties of BSL used in Glasgow, Newcastle, Manchester, London and Bristol. Regional differences were seen in culturally significant signs (e.g., ‘deaf’, ‘hearing’, ‘interpreter’), everyday lexical items (e.g., ‘British’, ‘business’, ‘theatre’) and forms new to the deaf community (e.g., ‘discrimination’, ‘community’). In some core semantic areas (such as colour terms, days of the week, and numerals), signs exhibited substantial regional variation. It was the case, however, that while there were regional differences, there was usually one variant recognised across all regions, suggesting gradual emergence of a national standard [2].

In contrast to the evidence of considerable traditional lexical variation in BSL, it has been claimed that ASL may have a relatively more standardised lexicon than other documented sign languages [59]. In their lexical variation study, Lucas and colleagues [2] found that of the 34 target concepts they studied, 27 included a variant that appeared in the data from all seven sites across the USA. Lucas et al. [2] suggested that historical patterns of ASL transmission account for the existence of widely shared variants. The residential schools in each of regions they studied all had direct or indirect links with the American School for the Deaf in Hartford, Connecticut, which had trained deaf graduates as teachers who then were sent out across the USA to establish new schools during the 19^th^ century, leading to the spreading of a single variety of ASL across the continent.

## Methodology

The lexical data in this paper was elicited as part of the BSL Corpus Project. Here we briefly introduce the BSL Corpus Project by outlining the sites of collection, participant characteristics and the methods used in data collection, coding and analysis (for more detail, see [60]).

### Ethics Statement

Participants in this research were all deaf with British Sign Language as their main/preferred language. Participants were all aged 16 or over. The University College London (UCL) Research Ethics Committee guidelines state that “young people aged 16–18 with sufficient understanding are able to give their full consent to participate in research independently of their parents and guardians” so no additional consent was obtained from those under 18. It cannot be assumed that members of this language community (deaf BSL users) have fluent or full comprehension of written English. Therefore the comprehensive information statement and consent form in written English which are required by the UCL Research Ethics Committee were translated into British Sign Language by local deaf fieldworkers and deaf researchers working on the project. Questions were clarified in person in BSL and consent obtained in writing. This project including the consent procedure was approved by the UCL Research Ethics Committee (project ID 0864/001). All individuals pictured in this manuscript have given written informed consent to publish their images.

### British Sign Language Corpus Project

The BSL Corpus Project, which began in 2008, was the first large-scale corpus project to be undertaken for BSL. The aim of the project was to create a corpus of elicited and spontaneous BSL digital video data from deaf native, near-native and early learners of BSL. The project has established an online, open-access video dataset available for researchers and the sign language community [60], and has provided data for a number of studies which have thus far investigated sociolinguistic variation and change, language contact and lexical frequency [61]–[62].

#### Sites

In order to obtain samples of regional variation, data were collected from eight sites across the UK: Belfast, Birmingham, Bristol, Cardiff, Glasgow, London, Manchester and Newcastle. These sites were selected because they are, or were previously, locations of a centralised school for deaf children, and because, as relatively large urban centres, it was assumed that they would provide a sufficiently large deaf community from which to recruit.

#### Participants

**Figure 2:**
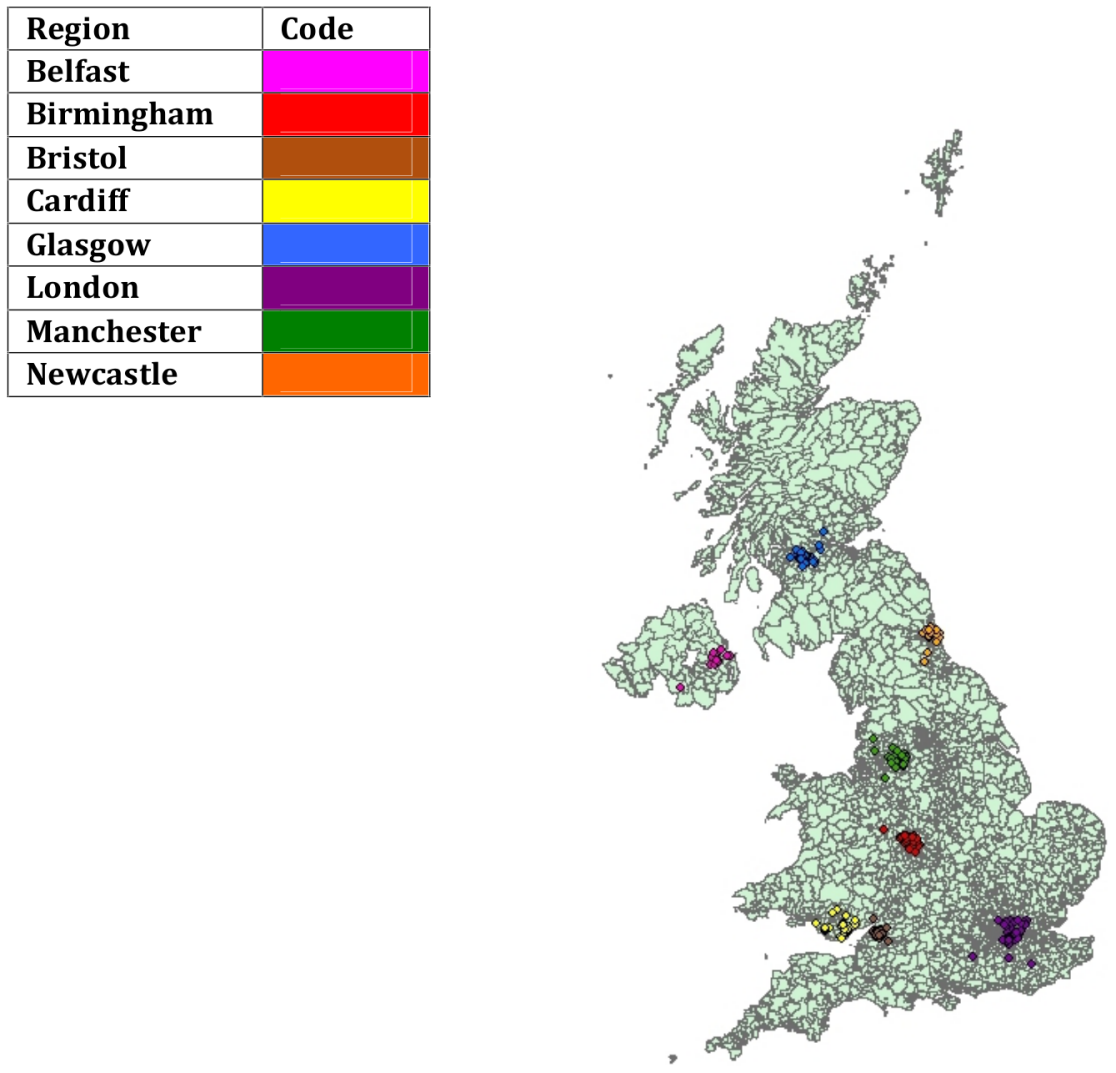
Regional distribution of the BSL Corpus Project participants.

Thirty participants were filmed at most sites, although slightly larger samples were collected in Bristol and London, with 32 and 37 participants respectively. In total, 249 deaf individuals were filmed. Figure 2 shows the regional distribution of the BSL Corpus Project participants, based on their home address at the time of filming. We attempted to recruit ‘lifelong’ users of BSL (cf., [2]) who were representative of the regional signs used in their particular region. Target participants were British-born, exposed to BSL before the age of seven, and had lived in the region where they were filmed for the previous 10 years, but a small number of people who did not fit these criteria were included. Five individuals were not British-born and 12 reported learning BSL after age seven (all but one, however, learned BSL before age 12). Participants were recruited by deaf community fieldworkers who were native or fluent BSL signers and familiar with the local deaf community. Fieldworkers recruited local deaf people they knew personally (e.g., friends, family, work colleagues) and who matched the project criteria. In recruitment we attempted to balance the sample for age groups, gender and social class and to represent deaf individuals from both deaf and hearing family backgrounds. Table 1 shows the participant characteristics in each site.

**Table 1:** Participant characteristics.

Sites	Total	Age			Gender		Ethnicity		Language background		Social class		School location (*some metadata missing)	
		Younger 16–39	Middle 40–59	Older 60+	Female	Male	White	Other	Deaf	Hearing	Working class	Middle class	Local	Non-local
Belfast	30	10	12	8	17	13	30	0	7	23	26	4	21	9
Birmingham	30	12	9	9	13	17	27	3	12	18	16	14	21	9
Bristol	32	9	14	9	17	15	30	2	17	15	16	16	16	16
Cardiff	30	10	12	8	17	13	28	2	6	24	22	8	10*	19*
Glasgow	30	10	13	7	15	15	27	3	6	24	17	13	20	10
London	37	8	19	10	17	20	31	6	13	24	15	22	20*	16*
Manchester	30	12	6	12	16	14	27	3	8	22	23	7	13*	16*
Newcastle	30	6	11	13	17	13	29	1	7	23	19	11	18*	11*
TOTAL	249	77	96	76	129	120	229	20	76	173	154	95	139*	106*

#### Data collection

The methodology for the BSL Corpus Project was based on two similar large-scale investigations of ASL [2] and Auslan [54] with some key differences. Unlike the other projects where participants were filmed in groups, all British participants were filmed in pairs with a person from the same region and of a similar age (in London, one participant was filmed a second time with a different partner). Four types of data were collected: a personal experience narrative, a free conversation of 30 minutes, responses to interview questions and responses to a lexical elicitation task.

**Figure 3:**
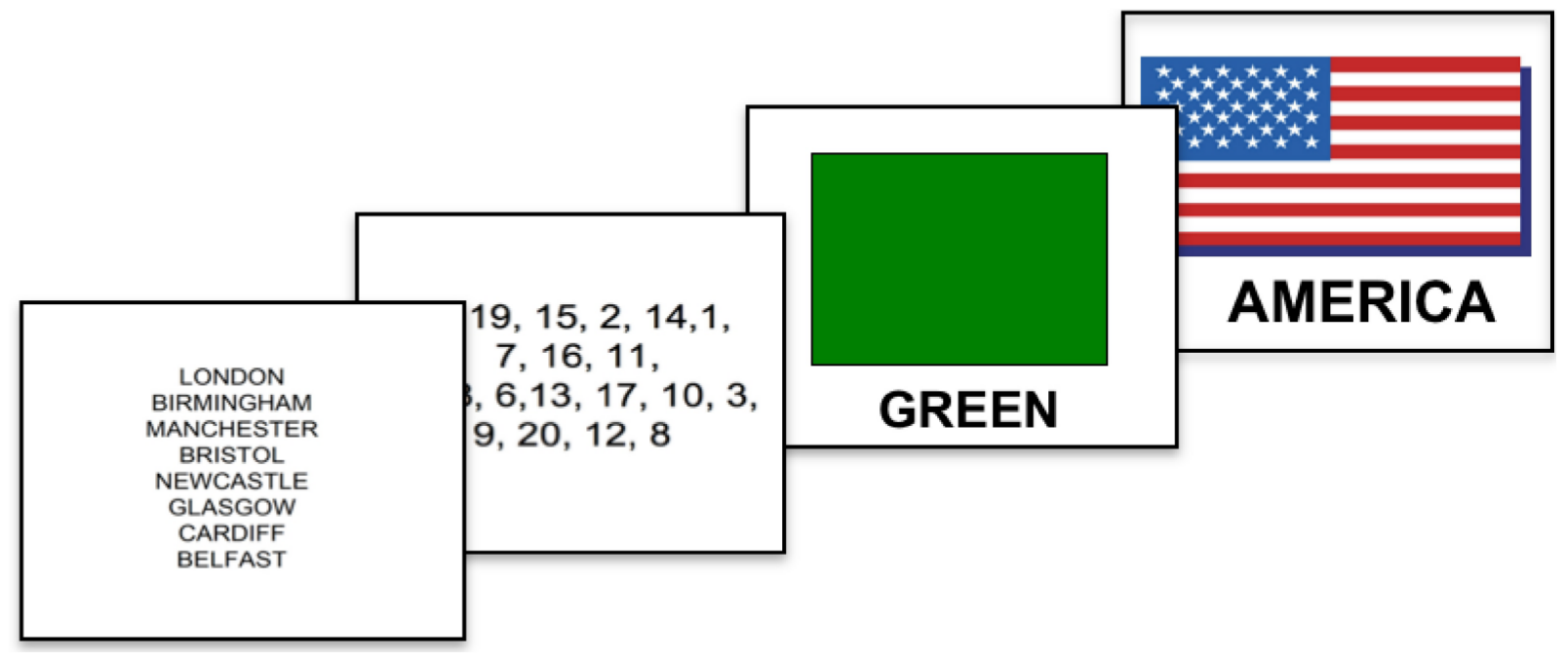
Example of the stimuli shown to participants.

In the lexical elicitation task, fieldworkers showed participants PowerPoint slides or flashcards for 102 concepts. Each slide displayed an image of the referent or something associated with it, and the equivalent English word underneath (see Figure 3 for examples). The concepts chosen were based on previous BSL lexical variation studies, existing dictionaries, and also following suggestions from the BSL Corpus Project Deaf Advisory Group [58],[63]. Responses for 41 of the 102 items are analysed here: five colour terms (‘brown’, ‘green’, ‘grey’, ‘purple’, ‘yellow’); eight countries (‘America’, ‘Britain’, ‘China’, ‘France’, ‘Germany’, ‘India’, ‘Ireland’, ‘Italy’); the numerals one to twenty; and eight UK place names (‘Belfast’, ‘Birmingham’, ‘Bristol’, ‘Cardiff’, ‘Glasgow’, ‘London’, ‘Manchester’, ‘Newcastle’). These 41 items were chosen as they represent the complete set of signs elicited for the four domains; colours, countries, numbers and UK place names. The specific signs were selected on the basis of existing lexicographical work on BSL, as well as a result of suggestions from the BSL Corpus Project Deaf Advisory Group (http://www.bslcorpusproject.org/team/). Numbers and colours are known to be highly variable according to region. Countries are believed to be a semantic field affected by lexical change and UK place names are believed to represent examples of exonymy/endonymy.

Stimulus items were also selected in order to investigate anecdotal reports about variation and change in their usage. Observation suggests that signs for country names, for example, have been undergoing considerable change in recent years. For UK placenames, anecdotal reports from BSL teachers indicated that lexical variants differ according to in-group and out-group membership.

For each target concept, participants were asked to produce the sign variant they use most on a daily basis. One limitation of this task is that sign variants are elicited in isolation and signers may respond to normative pressures or perceptions about what is appropriate for their region rather than offer the sign they actually use most. Therefore, a subset of the conversational data was also analysed to investigate whether the sign variants from the lexical elicitation task correspond to those used in the free conversation data (collected prior to the lexical elicitation task).

#### Data Coding (Signs for colours, countries & numbers)

Lexical variants for colours, countries and numbers (33 concepts) were elicited from 249 participants, producing a total of 8, 217 tokens. All tokens were annotated in ELAN [64]. Many participants produced multiple examples of signs and, as a result, either the variant stated to be the sign, or if not stated, the first variant produced, was coded. A small number of participants omitted or incorrectly identified some target concepts (e.g., one participant produced a sign meaning ‘eighteen’ in response to ‘16’ on the slide). For this reason, 18 tokens were excluded from the coding process. Our dataset included only one lexical variant for the numerals ‘one’, ‘two’ and ‘five’. As a result, these number signs were also excluded from the coding process, representing 747 tokens in the dataset. A further 120 tokens were excluded as some essential demographic information was missing from individual participants' background questionnaires, and thus social factors for those participants could not be coded. In total, 7332 tokens were analysed.

Phonological variants were grouped together as one lexeme [65]–[66]. Following previous work [1],[65], variants which were formationally related and differed only in one parameter (handshape, location, movement, orientation, or non-manual features) were considered to be phonological variants.

The lexical elicitation task produced an extremely complex dataset in which each of the individual stimuli represent a variable that could be investigated in detail. As a means to capturing overall patterns in the data, we coded each response as either a ‘traditional’ or ‘non-traditional sign’ for the signer's region. This would make it possible to investigate whether there is any evidence that levelling is taking place in BSL, and whether or not this language change is more strongly associated with any specific subgroup(s) (e.g., young males) in the British deaf community. The traditional signs for colours, countries and numbers for each region were determined by two methods. First, there are existing teaching resources about BSL lexical variation that make claims about the association of certain BSL signs with particular regions in the UK. For example, the lexical variant america4 (shown in Figure 1) has been claimed to be traditionally associated with the London/south-eastern region of England [67]. In addition, earlier studies in BSL describe a number of regional signs [68]. Secondly, the signs produced by elderly signers in each region were examined and each local deaf community fieldworker was consulted to confirm which of these represented traditional signs in their region (usually this was the most frequent variant in the data from the older signers). In cases where the actual productions of the oldest group of signers contradicted what signs were claimed to be traditional for the region by the fieldworker, these examples (a total of 610 tokens) were then excluded from the analysis.

Each token was coded for the following social factors: age, gender and social class of the signer. For this study, participants were categorised into three age groups: 16–39 years (younger), 40–59 years (middle) and 60 years and above (older) to reflect the different educational policies experienced by deaf children throughout the twentieth century (and thus our age groups reflect emic criteria [69]). Most of the participants in the older category would have attended residential schools and been educated predominantly using lip-reading and fingerspelling [13]. Those in the middle age group would have experienced an increasing emphasis on speech and lip-reading and the beginning of mainstreaming of deaf students into schools with hearing children. Younger signers are more likely to have attended mainstream schools with communication support workers, or experienced the shift to bilingual education following the increasing acceptance of BSL as a language. For social class, participants were classified as either working or middle class based on occupation and/or education (i.e., ‘working class’ referred to individuals working in unskilled, semi-skilled or skilled manual jobs, while ‘middle class’ were those who had a university education and/or worked in skilled non-manual jobs or professional/managerial positions). Whether an adult signer grew up signing from birth or acquired sign language later in life has been found to be an important predictor of lexical variation in sign languages [2]. Responses were thus coded as being from participants with hearing or deaf language backgrounds according to their parents' audiological status (whilst the assumption here is that only participants with deaf parents will have learnt sign language natively, this is not always the case as some deaf parents may be non-signers and some hearing parents may be fluent sign language users). Finally, the regional background of each signer was also included in the analysis. In Quinn's [23] study on BSL regional variation (unlike previous studies of sociolinguistic variation and change in sign languages), a signer's regional background was determined by the location of the school they attended rather than the region of the UK in which they lived at the time of data collection. For the purposes of the current study, participants' responses were coded both for their region of residence and their school location. A participant's school location was classified as ‘local’ if he or she had attended a school at any point in their education (primary and/or secondary school) located in the region in which they were currently living.

In addition to these social factors, the semantic category of the elicited sign (signs for countries, numbers or colours) was coded to see whether subcategories of signs were changing at a different rate than others.

In summary, sign variants produced for colours, countries and numbers were coded as traditional or non-traditional for the signer's region. The following social factors were investigated: region (Belfast, Birmingham, Bristol, Cardiff, Glasgow, London, Manchester, Newcastle), age (16–39, 40–59, 60+), gender (female, male), social class (middle, working), language background (deaf, hearing), school location (local, non-local). The semantic category of the sign was also coded (colour, country, number).

#### Data coding: sub-sample (UK placenames)

The UK placename data were analysed separately to investigate anecdotal claims about their usage. Such claims suggest that place name signs may work to index local, in-group versus non-local, out-group identity. For example, it is claimed that Bristol signers use a different lexical variant for ‘Bristol’ than those living elsewhere. To investigate this claim, fieldworkers from each of the eight regions were asked to identify which lexical variants were considered to be the local variants. The elicited forms for these UK placenames were analysed and the variants produced were coded as either local or non-local for the particular placename. In most cases, the local sign is a lexicalised form of fingerspelling consisting of the first letter and possibly some subsequent letters (e.g., the Manchester variant meaning ‘Manchester’ is formed by producing the manual letter ‘M’ followed by ‘C’) (for more discussion of lexicalised fingerspelling, see [70]–[71]). This is also the case for the following placenames: ‘Birmingham’ (B-H-M), ‘Bristol’ (B-L), ‘Cardiff’ (C-F-F), ‘Glasgow’ (G-W), and ‘Newcastle’ (N-C). For the placenames ‘London’ and ‘Belfast’, a different sign unrelated to fingerspelling is used locally. The data were analysed to investigate if there was a correlation between the use of a variant local to the region and participants' place of residence.

#### Data coding: sub-sample (Conversational data)

The effects of the observer's paradox were likely to be greater during the lexical elicitation task than the conversational task, due to the relatively greater attention to sign language production in the former compared to the latter activity [5]. Annotation work on the conversational data is ongoing, and only a subset of 500 tokens from 50 signers in Birmingham and Bristol have been annotated, making a total of 25,000 searchable tokens [72]. In this dataset, we searched for tokens of those colour, numeral, and country lexical items elicited in the lexical elicitation task to investigate whether participants produced identical or different lexical variants in the two datasets. In total, 570 different tokens were identified and analysed.

#### Data Analysis

For the current study, we carried out multivariate statistical analyses of the data using Rbrul [73]. Like the program GoldVarb, developed by [74], Rbrul can quantitatively evaluate the influence of multiple factors on variation. In addition, Rbrul uses mixed-effects modeling to group individual responses accounting for the effects of individual differences [75]–[76].

## Results

The results for the colour, country and number signs study are discussed first. This study investigates the relationship between the use of traditional regional signs and social factors (e.g., signers' age, gender, etc.). Following this, the results for the UK placenames study will be presented. Finally, the comparison between the conversational and lexical elicitation data will be discussed.

### Variables of analysis: Social factors

Of the 6722 tokens analysed, 5279 (79%) were classified as traditional for the signer's region. Participant and lexical item were included in the analysis as random effects. Table 2 presents the results, including the log odds, number of tokens analysed, percentage of traditional variants and the centred weight for each factor (with the use of traditional sign variants as the application value). Results with a positive log-odd and a factor weight over 0.5 (shown in bold) indicate that this factor results in an increased likelihood that the traditional variants will be used while a negative log-odd and a factor weight below 0.5 indicate an increased likelihood that non-traditional variants will be found in the data.

**Table 2:** Multiple logistic regression results for signs for colours, countries and numbers.

Factor Group	Factor	Log odds	Tokens	% of traditional signs	Centred weight
*Age (in years)	**60+**	**0.815**	**2042**	**87.6**	**0.693**
	**40–59**	**0.154**	**2606**	**81.4**	**0.538**
	16–39	−0.969	2074	66.1	0.275
*School location	**Local**	**0.281**	**3798**	**81.3**	**0.570**
	Non-local	−0.281	2924	75.0	0.430
*Language Background	**Deaf**	**0.223**	**2099**	**78.1**	**0.556**
	Hearing	−0.223	4623	78.7	0.444
Semantic category	**Colours**	**0.420**	**1222**	**83.6**	**0.603**
	**Numbers**	**0.006**	**3877**	**79.4**	**0.501**
	Countries	−0.426	1623	72.6	0.395
Social class	**Middle**	**0.09**	**2599**	**77.6**	**0.522**
	Working	−0.09	4123	79.1	0.478
Gender	**Male**	**0.018**	**3221**	**78.0**	**0.505**
	Female	−0.018	3501	79.0	0.495

Application value: Traditional signs.

*Factor groups significant at p<.05. 6722 tokens.

Input probability = 0.866, Mean = 0.785, Intercept = 1.868, Deviance = 5752.805. Random (participant) standard deviation = 1.064. Random (lexical item) standard deviation = 0.809.

We tested for interactions between the seven variables under investigation and found that region and school location were not independent of each other (region/school, p>0.05); as a result, we excluded region of residence from the analysis. Of the six factor groups remaining, the following three, in order from greater to lesser importance, predict the use of traditional signs: age, school location and language background. Participants in the older age group strongly favour the use of traditional signs (0.693), while those in the younger age group strongly disfavour the use of traditional signs (0.275). Those who were educated locally slightly favour the use of traditional signs (0.57) compared to those who were educated outside of the region where they reside (0.43). The third most significant predictor was language background, with participants with hearing parents slightly disfavouring the use of traditional signs (0.444). Participants with deaf parents slightly favour the use of traditional signs (0.556). The semantic category of the sign, social class and gender were not found to be significant. The results for each category (in increasing order of their proportion of traditional signs: signs for countries, numbers and colours) were analysed separately to look at the patterns of traditional sign use.

#### Signs for countries

In the country names dataset, a total of 1623 tokens were analysed. Table 3 presents the results. Language background, school location, social class and gender were not significant factors. Age was found to be an important factor, however. Older signers favoured the use of traditional signs (0.635) and younger signers disfavoured the use of traditional country signs (0.303).

**Table 3:** Multiple logistic regression results for signs for countries.

Factor Group	Factor	Log odds	Tokens	% of traditional signs	Centred weight
*Age (in years)	**60+**	**0.555**	**492**	**80.9**	**0.635**
	**40–59**	**0.278**	**630**	**77.0**	**0.569**
	16–39	−0.832	501	58.9	0.303
Language Background	**Deaf**	**0.212**	**515**	**73.4**	**0.535**
	Hearing	−0.212	1108	72.2	0.499
School location	**Local**	**0.165**	**916**	**74.2**	**0.541**
	Non-local	−0.165	707	70.4	0.459
Social class	**Working**	**0.005**	**1003**	**73.5**	**0.501**
	Middle	−0.005	620	71.1	0.499
Gender	**Female**	**0.04**	**841**	**74.0**	**0.51**
	Male	−0.04	780	71.1	0.49

Application value: Traditional signs.

*Factor groups significant at p<.05. 1623 tokens.

Input probability = 0.793, Mean = 0.726, Intercept = −1.342, Deviance = 1646.468. Random effects (participant) standard deviation = 0.809. Random effects (lexical item) standard deviation = 1.131.

#### Signs for numbers

Age, school location and language background were significant predictors of the use of traditional number signs (see Table 4). Older signers strongly favoured the use of traditional number variants (.778) and younger signers strongly disfavoured the use of traditional number variants (.211). A chi-square analysis revealed a significant difference (χ^2^ = 49.53, p<0.001) in the use of traditional variants between the younger and middle age groups but no significant difference between the middle and older age groups (χ^2^ = 0.857, p = 0.835). School location was the second most significant factor. Signers who attended a school in the same region in which they currently reside favour the use of traditional number signs (.601) to a greater extent than signers who attended a school outside of their region (.399). Signers from a hearing family slightly disfavour the use of traditional number signs (.418) while signers with deaf parents slightly favour the use of traditional number signs (.582). Social class and gender were not found to be significant.

**Table 4:** Multiple logistic regression results for signs for numbers.

Factor Group	Factor	Log odds	Tokens	% of traditional signs	Centred weight
*Age	**60+ years**	**1.256**	**1181**	**90.3**	**0.778**
	**40–59 years**	**0.060**	**1501**	**81.3**	**0.515**
	16–39 years	−1.316	1195	66.3	0.211
*School location	**Local**	**0.408**	**2195**	**82.7**	**0.601**
	Non-local	−0.408	1682	75.2	0.399
*Language Background	**Deaf**	**0.33**	**1194**	**79.0**	**0.582**
	Hearing	−0.33	2683	79.6	0.418
Social class	**Middle**	**0.169**	**1506**	**78.6**	**0.542**
	Working	−0.169	2371	80.0	0.458
Gender	**Male**	**0.1**	**1849**	**79.2**	**0.525**
	Female	−0.1	2028	79.6	0.475

Application value: Traditional signs.

*Factor groups significant at p<.05. 3877 tokens.

Input probability = 0.909, Mean = 0.794, Intercept = 2.301, Deviance = 3018.346. Random (participant) standard deviation = 1.654. Random (lexical item) standard deviation = 0.697.

#### Signs for colours

Of the 1222 colour tokens analysed, only 16% (201 tokens) were non-traditional sign variants. The signer's age and school location were important in predicting the use of traditional colour signs (see Table 5). Gender, language background and social class were not significant predictors for the lexical variant chosen. Similar to results for the other semantic categories, older signers showed a preference for the use of traditional forms (0.594) compared to younger signers (0.334). Also, those signers who were educated locally favoured the use of traditional signs (0.556) while those who were educated outside of the region slightly disfavoured the use of traditional signs (0.444).

**Table 5:** Multiple logistic regression results for signs for colours.

Factor Group	Factor	Log odds	Tokens	% of traditional signs	Centred weight
*Age (in years)	**60+**	**0.380**	**369**	**87.5**	**0.594**
	**40–59**	**0.312**	**475**	**87.2**	**0.577**
	16–39	−0.692	378	75.1	0.334
*School location	**Local**	**0.224**	**687**	**86.0**	**0.556**
	Non-local	−0.224	535	80.4	0.444
Gender	**Male**	**0.045**	**590**	**83.4**	**0.511**
	Female	−0.045	632	83.7	0.489
Language background	**Deaf**	**0.035**	**390**	**81.8**	**0.509**
	Hearing	−0.035	832	84.4	0.491
Social class	**Middle**	**0.032**	**473**	**82.9**	**0.508**
	Working	−0.032	749	84.0	0.492

Application value: Traditional signs.

*Factor groups significant at p<.05. 1222 tokens.

Input probability = 0.885, Mean = 0.836, Intercept = 2.037, Deviance = 990.857. Random effects (participant) standard deviation = 0.898. Random effects (lexical item) standard deviation = 0.682.

#### Signs for UK placenames

To investigate anecdotal reports that individuals who reside within a given urban centre use a different sign variant to refer to that city than non-residents do, a total of 1992 tokens were classified as either local or non-local for the particular placename analysed. The results revealed that, with the exception of ‘Glasgow’, ‘London’ and ‘Manchester’, the use of the local placename variant significantly correlated with residency in that location, with residents found to strongly favour the use of the local variant: Belfast (>0.999), Birmingham (0.673), Bristol (0.736), Cardiff (0.724) and Newcastle (0.794). Table 6 presents the results of each significant Rbrul run for the UK placenames data including the log odds, the number of tokens and the centred weight.

**Table 6:** Multiple logistic regression results for UK placenames.

Lexical item	Factor	Log odds	Tokens	Centred Weight
Belfast	**Residents**	**8.395**	**30**	**>0.999**
	Non-residents	−8.395	219	<0.001
Birmingham	**Residents**	**0.72**	**30**	**0.673**
	Non-residents	−0.72	219	0.327
Bristol	**Residents**	**1.024**	**32**	**0.736**
	Non-residents	−1.024	217	0.264
Cardiff	**Residents**	**1.017**	**30**	**0.724**
	Non-residents	−1.017	217	0.266
Newcastle	**Residents**	**1.347**	**30**	**0.794**
	Non-residents	−1.347	219	0.206

Application value: Local variant for region. All factor groups significant at p<.05.

### Extent of lexical variation in BSL

This study has investigated the lexical variants produced by 249 signers for 41 concepts within the semantic fields of colours, numerals, countries, and UK placenames. In total, 10, 209 tokens were elicited. Of these, 295 separate lexical variants were identified for the 41 stimuli. Thus, the variation for these concepts in BSL is considerable, mirroring the findings of previous studies [58]. This was most true of the signs for colours: we found 22 variants for ‘purple’. It is likely that this range is not exhaustive, given that it is based on data collected in only eight sites across the UK. Importantly, 79% of responses (5279 tokens) maintained the use of traditional signs for each region, suggesting that any loss of regional variation in this part of the BSL lexicon is limited. Some semantic fields are, however, undergoing greater loss of variation than others. Only 72% of signs for countries (1178 tokens) were traditional variants, compared to 79% of signs elicited for numbers (3080 tokens) and 84% of signs for colours (1021 tokens). Table 7 shows the number of lexical variants per item: over half of the items investigated (21 lexical items) exhibited eight or more lexical variants. Six of the stimuli have ten lexical variants and three have more than thirteen lexical variants (‘grey’, ‘purple’, ‘thirteen’).

**Table 7:** Number of lexical variants per concept.

	Number of stimuli							
Number of variants	0	1	2	3	4	5	6	7
1				x				
2		x						
3			x					
4				x				
5				x				
6				x				
7						x		
8								x
9				x				
10							x	
11			x					
12	x							
13+				x				
TOTAL								

#### Conversational data

To consider the use of traditional signs across settings all examples of the signs for colours, countries and numbers from the LFS conversational data were compared to those elicited as part of the lexical elicitation task. A total of 570 tokens were coded for analysis. Of these, 124 tokens (22%) were not the same sign variant as those elicited during the lexical elicitation task, suggesting that 78% of elicitations as part of the lexical elicitation task were an accurate representation of the signer's actual lexical use when there is less attention paid to their language production. There were 26 instances in which a response involved a signer using a traditional variant in one setting and another traditional variant in a different setting, or a non-traditional variant in one setting and a different non-traditional variant in another setting. Twelve percent of responses (15 of 124) involved the use of a non-traditional variant in the lexical elicitation task and a traditional variant in the conversational task. In sixty-seven percent of responses (83 of 124) a signer used a traditional variant in the lexical elicitation task and a non-traditional variant in the conversational task. Overall, the results indicate that the majority of signers used the same variant across different settings (conversation and the lexical elicitation tasks). In conversation, the more naturalistic setting, a minority of participants produced more non-traditional variants.

## Discussion

### Variation according to social factors and semantic category

Participants' use of traditional signs was conditioned by a number of social factors, in order of significance: age, school location, and language background. In this section, we discuss these findings in relation to previous sign language research, explore whether the results reveal language change in progress, and finally suggest what this study can tell us about the BSL variation in the future.

The results suggest that age is the most significant factor predicting the use of traditional signs with a decline in use from older to younger signers. This was true, to differing degrees, across all regions. Some variants were found to be unique to certain age groups. For example, some number sign variants which originate from Irish Sign Language were not present at all in the data elicited from younger participants. These variants are associated with St. Vincent's School for deaf children in Glasgow, a Catholic school in which teachers used Irish Sign Language as the language of instruction until the 1950s [56]. This finding suggests that these variants may, with time, disappear from the BSL lexicon.

School location is the second most important factor predicting the use of traditional signs in BSL. Those individuals who were educated locally in our study used a higher proportion of regional signs than those individuals who had attended a school outside the region. The findings do suggest that, although individuals may have lived in a given region for the last 10 years, they do not entirely adapt their lexicon to the local variety. This finding appears to confirm the importance of schooling in the maintenance of regional variation in BSL, supporting Quinn's [23] findings that the BSL variants acquired at school strongly influence the variants used in adulthood. It also suggests that the geographical location of a participant's school might be a better predictor of lexicon in adulthood than current region of residence.

Language background is also a significant factor in predicting the use of non-traditional signs. Signers with deaf parents favour the use of traditional signs, supporting previous work on ASL which found that signers with deaf parents favour the use of ‘conservative’ variants [2]. The significance of language background is not surprising given that our age-related findings indicated that older signers use more traditional forms. It is likely that deaf children learning BSL from deaf parents will be exposed to a higher proportion of traditional variants compared to their counterparts from hearing families (since most hearing parents have not learned BSL before the birth of their deaf child). These findings highlight the importance of deaf native signers in maintaining and transmitting BSL regional variation.

Although semantic category was not a significant factor in predicting the use of traditional signs, the results indicate that some semantic categories are undergoing greater change than others. In this study, signs for countries are changing at a faster rate than signs for numbers and colours.

#### Signs for countries

Signs for countries appear to have undergone the most dramatic change. Around half of the responses by younger participants in the lexical elicitation task (302 tokens) were not traditional signs for their region. Younger and older signers may have adopted a different lexical variant later in life from the variant acquired in their early years. This is quite evident from the discussions between participants during the lexical elicitation task in which many older and younger signers explicitly mention the sign they formerly used and the sign they use now.

**Figure 4:**
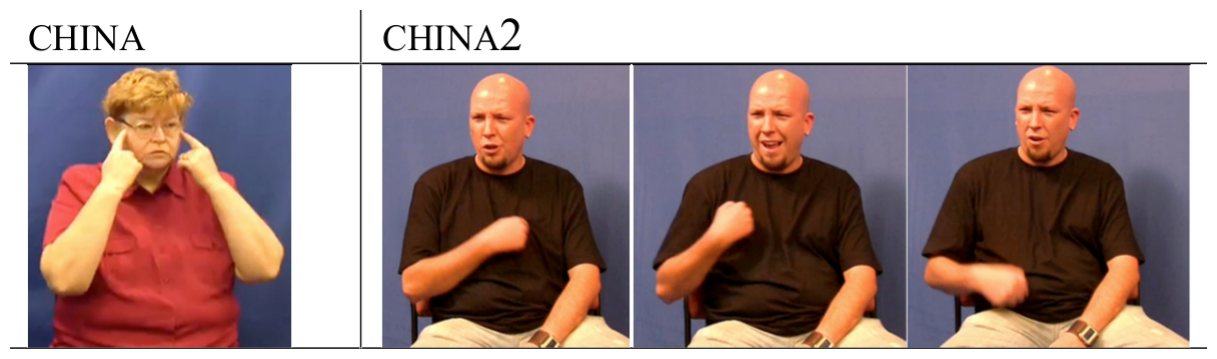
Examples of variants for ‘China’ in BSL.

By comparing the traditional variant for each region to the most frequent non-traditional variant used amongst younger signers, we can observe the direction of change in some country name signs. For example, the traditional BSL sign meaning ‘China’ is produced at the eyes with a twist of the wrist of both hands (see china, Figure 4). The use of this sign has been perceived by some both within the deaf community and outside it as ‘politically incorrect’, presumably because the sign depicts the characteristic eye shape of east Asian people [35]–[36]. The traditional variant glossed as china, shared across all eight regions in the BSL Corpus dataset, is produced by 67% (164 participants) overall, although younger signers produce only 12% of this variant. Instead, a variant glossed as china2 (Figure 4), which portrays an aspect of the country's national costume, is the most frequent non-traditional variant amongst younger signers. The sign variant china2 was reported by Sutton-Spence and Woll [13] to have been introduced into BSL as a ‘politically correct’ alternative and is used by 61% of the younger corpus participants (47 participants) compared with only 24% of the older signers (18 participants).

**Figure 5:**
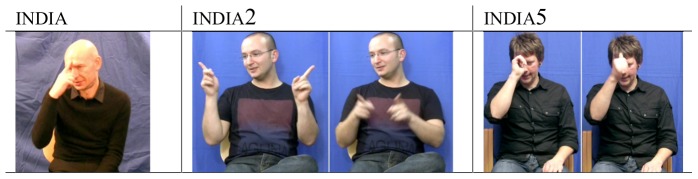
Examples of sign variants for ‘India’ in BSL.

The change in progress for signs meaning ‘China’ appears to be the same across all eight regions in the BSL Corpus Project. This shift in signs representing China co-occurred with the introduction of signs for other east Asian countries. The pattern of change (from a sign representing physical characteristics to one representing clothing) is not observed for other country name signs. For example, the BSL variant india (Figure 5) is the traditional variant for ‘India’ in five of the eight collection sites of the BSL Corpus Project. This variant is produced at the forehead to represent the bindi or tilak worn by followers of the Hindu religion in India. This sign is perceived as inappropriate by some signers (see [13]), as it is seen to exclude Indian Muslims. In these five regions, 40% of participants (n = 75) do not report using this traditional sign, instead using india2 (Figure 5), which is thought to depict the shape of the country. Of this 40%, 21% (n = 39) are younger signers, 10% (n = 19) are middle-aged signers, and 9% (n = 17) are older signers. In the remaining three regions, where the sign variant india is not traditional, a similar proportion of participants are not using their traditional regional sign (38%, n = 23). Despite this, however, the most frequent non-traditional variant (used by 85%, n = 23 of participants) is india. This suggests that political correctness does not explain all examples of language change in this component of the BSL lexicon.

Additionally, a small number of signers in the younger and middle groups are using a sign variant borrowed from Indian Sign Language [77]. It is interesting that this variant (india5, Figure 5), referring to the same iconic feature as india, is spreading while the traditional BSL sign appears to be falling out of favour. Thus the process of lexical change for this particular example is complex, and we could speculate that because of concerns about excluding Muslims (as noted above), some signers adopt the newer sign india2, while others prefer to use lexical borrowing. This is consistent with work by Lucas and colleagues, [2] who suggested that similar changes in ASL were a means of showing respect for other cultures. Some signers may be making such choices consciously, following media coverage that has raised deaf community awareness of these changes (e.g., *See Hear* - [62]).

**Figure 6:**
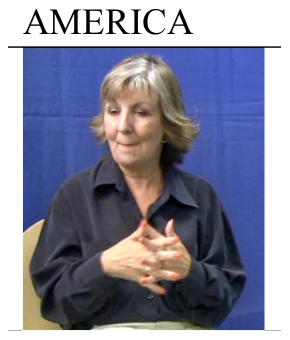
Example of borrowed variant for ‘America’.

Lexical borrowing may be the source of several of the country signs in this study as we have seen with ‘India’. Like ‘India’, the traditional variants for ‘America’ in all regions have been replaced amongst younger signers with the borrowed ASL form america (as in Figure 6). This can be seen in all regions except Belfast where america is already the traditional sign used amongst older signers (possibly reflecting known earlier language contact with ASL). Similarly in the case of ‘Germany’, lexical borrowing may have occurred much earlier than signs for the other countries, given that the sign used in DGS (Deutche Gebärdensprache, German Sign Language) to represent ‘Germany’ is also used by the oldest generation of BSL signers from four regions in the BSL Corpus dataset.

**Figure 7:**
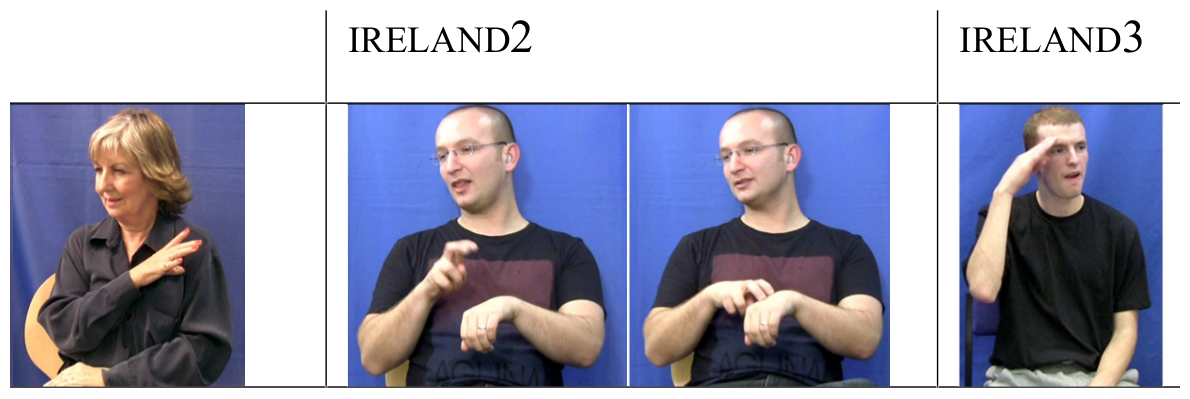
Examples of the sign variants for the concept ‘Ireland’ in BSL.

Finally, not all borrowed forms may come from the corresponding country's sign language. For example, we find that seven regions (again, except for Belfast which already uses ireland2, see Figure 7) share the traditional variant ireland produced with a flicking movement at the chest area (a popular anecdotal explanation for this sign is that it represents a shamrock). However, of these seven regions we find that 81% of signers (n = 44) who do not use the traditional variant, instead using a variant associated with ASL (see ireland2, Figure 7). Younger signers do not show any preference for ireland3, a form borrowed from Irish Sign Language (ISL).

To summarise, signs for countries in BSL have experienced rapid lexical innovation. This may, in some cases, be linked to pressures from political correctness but it may also reflect increased contact between deaf communities around the world leading to the borrowing of signs. The language change processes for signs for numbers and colours will be discussed in the next section.

### Language change & levelling

#### Signs for numbers and colours

Previous research has suggested that signs acquired first are generally maintained over a signer's lifetime [23]. The use of a high proportion of traditional signs for colours and numbers was also correlated with having been educated locally (unlike signs for countries, for which school location was not significantly associated with use of traditional signs), reinforcing the observation that signers maintain their school variants over the course of their lifetime. We adopt here the apparent time hypothesis to interpret the age-related results. A decrease in traditional number signs in younger age groups is an indicator that levelling may be taking place [11]. With number signs, younger signers appear to be adopting the two systems used in southern England (London or Bristol), both of which are widely known. Our levelling results resemble the findings of McKee and McKee [14] for NZSL. They concluded that there was evidence of increasing standardisation towards Australasian Signed English variants following the introduction of this signed English system in New Zealand deaf education from 1979. While the reasons for levelling in the current study remain unexplored, one possible cause for the rapid change is the loss of transmission following the closure of deaf schools.

In the dataset presented here, we have analysed the changes in colour signs as well as number signs. Results must be viewed with caution as the colour dataset is significantly smaller than the number dataset (17 target numeral stimuli compared to 5 target colour stimuli), but the results indicate that there have been fewer changes in colour signs than number signs. Age was found to be a contributing factor in the use of colour signs with younger signers using a decreasing proportion of traditional signs compared to older signers. Instead, those younger signers who used non-traditional signs used variants associated with the London region (e.g., brown3); variants associated with more than one region (e.g., green1 in Manchester and London, grey1 in Birmingham and London); or single manual letter forms, where the first letter of the corresponding lexical item in English is fingerspelled (e.g., grey1, purple1 and yellow1 are produced by using the fingerspelled letters ‘g’, ‘p’ and ‘y’ respectively, and in some cases modifying the movement). Overall, the patterns suggest that younger signers are using a variant that is widely used and/or reflects English influence.

#### Signs for UK placenames

With UK placenames, we were interested in the folk belief that residents of a city use a different sign variant for their city's name than non-residents. The only exceptions were the signs for ‘Glasgow’, ‘London’ and ‘Manchester’: almost all signers in all regions used the same lexical variant for ‘Glasgow’ (221 tokens), ‘London’ (244 tokens) and ‘Manchester’ (209 tokens). In most cases, the endonym, or local name for the city, was a reduced fingerspelled form, as described above, with the exception of the sign for ‘Belfast’. The exonym, or name used for the city by individuals from outside that city, in some cases was a calque – i.e. a literal translation of the equivalent English words. For example, one exonym variant for ‘Manchester’ consists of a compound of individual signs man and chest. Other exonyms include the use of signs pistol or petrol to refer to ‘Bristol’, perhaps because of similarities in the mouthing of these English words.

## Conclusion

In this investigation of lexical variation and change in signs for 41 key concepts in BSL, we have identified a number of processes taking place in the language that reflect different conditioning factors operating on different subsets of the data. Age is an important predictor of lexical variation and change across all groups of signs in this study. Signs for countries are subject to a number of external influences, including political correctness, changing attitudes towards lexical borrowing, and greater international mobility and transnational contact. The change we see here is age-graded, with anecdotal evidence of some older signers also adopting newly introduced variants. Changes in the use of traditional regional signs for colours and numbers, however, do not appear to be subject to changes in attitudes to language, but appear to reflect changes in the transmission of BSL as well as increased mobility within the UK and exposure to lexical variation in BSL via the media.

This descriptive and quantitative analysis of a large dataset of BSL provides a ‘snapshot’ of BSL lexical variation and change synchronically and suggests how societal changes have directly influenced BSL. This study lays the groundwork for more detailed ethnographic studies and investigations into the relationship between variation, change and language attitudes in the British deaf community.
